# Oxymatrine Protects Chondrocytes against IL-1*β*-triggered Apoptosis *in Vitro* and Inhibits Osteoarthritis in Mice Model

**DOI:** 10.1155/2022/2745946

**Published:** 2022-09-27

**Authors:** Diliyaer Mohetaer, Li Cao, Yang Wang

**Affiliations:** First affiliated hospital of Xinjiang Medical University, Urumqi, China

## Abstract

**Background:**

Osteoarthritis (OA) is a multifactorial disease with various risk factors, resulting in the degeneration of articular cartilage and whole joints. However, to date, no effective disease-modifying therapy for OA has been developed. Oxymatrine (OMT) is associated with many pharmacological effects, including anti-inflammatory, antiapoptotic, and antioxidative properties. However, the role of OMT in OA remains unclear.

**Materials and Methods:**

An IL-1*β*-induced chondrocyte model and anterior cruciate ligament transection (ACLT)-induced murine model of OA were constructed. The effect of OMT on chondrocyte viability was assessed using the CCK-8 assay. The protein level was assessed by Western blot analysis, and the apoptosis rate was assessed by flow cytometry in vitro and TUNEL staining in OA model mice. The effect of OMT on the degradation of articular cartilage in ACLT-induced OA mice was assessed by histological analysis.

**Results:**

OMT at 0–2 mg/mL showed no conspicuous cytotoxicity on chondrocytes after 24 hours of incubation. OMT at 0.5, 1, and 2 mg/mL inhibited IL-1*β*-triggered apoptosis, upregulated MMP13, MMP9, and Col X, and upregulated Col II in chondrocytes in vitro. OMT represses the NF-*κ*B signaling cascade in IL-1*β*-triggered chondrocytes in vitro. In an in vivo study, OMT decreased the apoptosis rate of chondrocytes and exerted a protective effect against the degradation of articular cartilage in ACLT-triggered OA mice.

**Conclusion:**

OMT plays a protective role against chondrocyte injury induced by IL-1*β* in vitro or ACLT in vivo. OMT may play a role in chondrocytes during OA by inhibiting NF-*κ*B signaling by decreasing the phosphorylation of p65 and I*κ*B. OMT treatment may be a promising chondroprotective approach to delay OA cartilage progression.

## 1. Introduction

Osteoarthritis (OA) is a multifactorial disease with various risk factors that result in the degeneration of articular cartilage and whole joints [1]. There is no effective disease-modifying therapy for individuals with OA [2], emphasizing the need for the rapid development of safe and effective therapies to manage the condition [3]. The normal functioning of healthy joints depends on the integrity of the extracellular matrix structure of articular cartilage [4]. Chondrocytes are unique cell types in the articular cartilage that function to maintain homeostasis by regulating anabolic and catabolic activities [5]. Under OA conditions, chondrocytes are stimulated by proinflammatory cytokines, which subsequently upregulate the expression of cartilage degradation-related enzymes and inflammatory cytokines, thereby interfering with articular cartilage homeostasis [6]. During the early stages of osteoarthritis development, there is a decline and elevation in the synthesis of type II and X collagen, respectively [7]. Consequently, this phenomenon causes degeneration of articular cartilage, suggesting that type X collagen is an indicator of chondrocyte hypertrophy [8]. Notably, chondrocytes undergo hypertrophy and dedifferentiation during osteoarthritis development, which causes a change in their metabolic activities, increases chondrocyte cell death, and causes production of the extracellular matrix with poor biomechanics, further aggravating tissue damage and diminishing joint function [9]. Previous studies have demonstrated that IL-1*β* can induce and activate the NF-*κ*B signaling cascade in chondrocytes, thereby remarkably affecting their metabolism and apoptosis [10]. Moreover, the NF-*κ*B signaling cascade plays a vital role in cartilage degradation during the process of OA [11]. Additional research has revealed that upregulation of inflammatory cytokines subsequently activates the NF-*κ*B signaling pathway via the formation of positive feedback, thereby aggravating chondrocyte apoptosis and the degradation of articular cartilage [12, 13]. Therefore, inhibiting inflammatory pathways, coupled with suppressing the apoptosis of inflammation-related chondrocytes, is considered an effective method for treating OA.

Oxymatrine (OMT), a natural alkaloid of tetracyclic quinolizidines, is extracted and purified from Sophora flavescens [14]. In fact, OMT has attracted considerable research attention in recent years owing to its effects on oxidative stress, inflammation, and apoptosis [15–19]. Recent investigations have revealed that OMT can inhibit NF-*κ*B signaling by blocking I*κ*B kinase-triggered I*κ*B phosphorylation, as well as by preventing I*κ*B degradation, thereby downregulating proinflammatory cytokines [20–22]. Other studies have demonstrated that OMT can inhibit cell apoptosis by regulating mitochondrial functions or via the mitochondrial signaling cascade [23]. Recently, OMT was found to effectively regulate the TLR/NF-*κ*B signaling pathway to upregulate levels of the antiapoptotic protein BCL-2 and suppress cell apoptosis, thereby promoting functional recovery after spinal cord injury in adult rats [24]. Based on these study findings, we hypothesized that OMT plays a role in the regulation of OA.

To test this hypothesis, we used a mouse model to explore the effects of OMT on IL-1*β*-triggered chondrocytes and ACLT-induced OA. In addition, we investigated the effect of OMT on the NF-*κ*B signaling pathway to elucidate the underlying mechanisms.

## 2. Materials and Methods

### 2.1. Materials

Oxymatrine was provided by Shanghai Macklin Biochemical Co., Ltd. (Shanghai, China), whereas PBS, FBS, penicillin-streptomycin, trypsin, and DMEM/F12 were acquired from Gibco Life Technologies. The CCK-8 kit was supplied by Beijing Solarbio Technology Co. Ltd. Antibodies specific for *β*-actin (Abcam, ^#^ab8227), Bax (Bioss, ^#^bs-0127R), type II collagen (Bioss, ^#^bs-11929R), Bcl-2 (Bioss, ^#^bs-0032R), type X collagen (Bioss, ^#^bs-20085R), cleaved-caspase-3 (Abcam, ^#^ab214430), cleaved-caspase-9 (CST, ^#^9507S), I*κ*B*α* (CST, ^#^9242S), p-I*κ*B*α* (CST, ^#^2859T), p65 (CST, ^#^8242T), p-p65 (CST, ^#^3303T), MMP-13 (Proteintech, ^#^18165-1-AP), and MMP-9 (Proteintech, ^#^10375-2-AP) were used in this study. Male SPF C57BL/6 mice aged 3 months, were purchased from Jiangsu Aniphe Biolaboratory Inc. Finally, C57BL/6 suckling mice aged 3 days, were provided by the Animal Experimental Center of Xinjiang Medical University, Xinjiang, China.

### 2.2. Methods

#### 2.2.1. Isolation and Culture of Murine Primary Chondrocytes

Primary chondrocytes were isolated from 3-day-old C57BL/6 suckling mice using type II collagenase (Gibco, USA) according to a standard protocol [25]. The isolated chondrocytes were inoculated into a DMEM/F12 medium enriched with 10% FBS and maintained in a cell incubator at 37°C with 5% CO_2_. The cells were then subcultured to 90% confluence. Expression levels of collagen type II and collagen type X mRNAs were analyzed via quantitative real-time PCR (qRT-PCR), and relative expression was computed using the comparative Ct approach to evaluate chondrocyte differentiation.

### 2.3. Cell Viability Assay

Cell viability was assessed using a CCK-8 assay kit (Solarbio, China), as described by the manufacturer. Briefly, cells were plated in 96-well plates at a seeding density of 4 × 10^3^ cells/well and then cultured with or without OMT (0.125, 0.25, 0.5, 1, 2, 4, and 8 mg/mL) for 24 hours. Subsequently, 10 *μ*L of CCK‐8 solution was added to each well, followed by a 1 hour incubation at 37°C. The absorbance in each well was measured using a Multiskan™ GO microplate reader (Thermo Fisher Scientific, USA) at 450 nm.

### 2.4. Determination of Cell Apoptosis

Suspended cells were labeled with Annexin V-APC and 7-AAD using the Annexin V-APC/7-AAD Apoptosis detection kit (Absin Bioscience Inc., China) and incubated at room temperature (RT) for 15 minutes. The rate of apoptosis was analyzed within 1 hour using a flow cytometer (Beckman, USA).

### 2.5. Quantitative Reverse Transcription-Polymerase Chain Reaction

Isolation of total RNA was performed using TRIzol reagent (Thermo Fisher Scientific, USA) from cultured chondrocytes after 24 hours incubation with IL-1*β* and OMT, as described by the manufacturer, and quantified using a nanodrop spectrophotometer. Samples with an A260/A280 ratio of ≥1.8 were used. cDNA was generated from 1 *μ*g of total RNA using the PrimeScript RT Master Mix kit (Takara Bio, Japan), followed by qRT-PCR analysis with the SYBR FAST qPCR Master Mix (Takara Bio, Japan), targeting specific genes whose primer sequences are listed in Table 1, the expression levels of the target genes were calculated using the comparative Ct method.

### 2.6. Western Blot Assay

Cells were collected after 24 hours of incubation with IL-1*β* and OMT, rinsed thrice in cold PBS, and pelleted by centrifugation at 1000 rpm for 5 minutes. Isolation of total RNA from the pellet was performed using RIPA buffer. Thereafter, protein quantitation was performed using a BCA assay. Equal quantities of protein samples were fractionated by SDS-PAGE and blotted onto polyvinylidene fluoride (PVDF) membranes (Merck Millipore). Membranes were blocked with 5% nonfat milk at RT for one-hour, and incubated overnight with diluted primary antibodies (*β*-actin 1 : 5000, Bax 1 : 1000, type II collagen 1 : 1000, Bcl-2 1 : 1000, type X collagen 1 : 1000, Cleaved-caspase-3 1 : 1000, I*κ*B**α** 1 : 1000, p-I*κ*B*α* 1 : 1000, p65 1 : 1000, p-p65 1 : 1000, MMP-13 1 : 1500, and MMP-9 1 : 1500) at 4°C. Thereafter, the membranes were rinsed in TBST and then incubated for two-hours with secondary antibodies (goat antirabbit HRP conjugated, 1 : 5000, Proteintech) diluted in TBST at RT. Finally, the immunoreactive bands were visualized with enhanced chemiluminescence (ECL) reagents (Biosharp, China).

### 2.7. Animal Experiments

All animal experimental protocols were approved by the Xinjiang Medical University Institutional Animal Care and Use Committee (protocol no. IACUC-20200924-27). Male SPF C57BL/6 mice aged 3 months were selected. All animals were housed in normal mouse cages, in a pathogen-free environment, with a 12 hour light-dark cycle. Access to food and water was unlimited. Prior to the study, all mice were acclimated to a new feeding environment for 3–4 days. Mice were sedated with pentobarbital (40 mg/kg) intraperitoneally and subjected to anterior cruciate ligament transection (ACLT) or sham surgery. Briefly, the right knee joint was exposed via an anterior incision of the knee joint, and the joint capsule was opened using the medial parapatellar method. Next, the patella was laterally dislocated, and the knee joint was fully flexed to expose the anterior cruciate ligament (ACL). Transection of the ACL was performed under a microscope and the procedure was completed with layer-by-layer suturing. The knee joint was initially exposed via a medial capsular incision and the surgical skin incision was closed with sutures for the sham procedure. After a 4-week preliminary experiment to determine the optimal OMT dosage, the mice were randomly allocated to one of six groups (*n* = 5): sham, vehicle, or various OMT concentrations (10, 20, 40, and 80 mg/kg). Preliminary experiments showed that 80 mg/kg OMT generated the best chondroprotective effect, while lower concentrations (10, 20, and 40 mg/kg) had a weaker effect. ACLT-induced OA mice in the OMT group were intraperitoneally inoculated with 80 mg/kg OMT for 8 weeks. Mice in the sham and ACLT-induced OA mice in vehicle groups were inoculated with a similar volume of saline. All mice in each group were sacrificed using an overdose of sodium pentobarbital 8 weeks after surgery.

### 2.8. Histological Analysis

Tissues from each animals' right knee joint were extracted and preserved for 24 hours in 10% buffered formalin, then decalcified for three weeks in 10% EDTA in 0.1 M phosphate buffer (pH = 7.4). The tissues were subsequently fixed in paraffin and cut into 4 *μ*m-thick sections using a microtome. For cartilage examination, each section was stained with hematoxylin-eosin (H&E) or safranin O/fast green (SF) and examined under a light microscope (Olympus, Japan). Based on the results of SF staining, the Osteoarthritis Research Society International (OARSI) score of articular cartilage was calculated for each group to assess the state of the articular cartilage and proteoglycan level [26]. Based on results of H&E staining, the thickness of hyaline cartilage (HC) and calcified cartilage (CC) was measured according to the position of the tidemark using Image Pro-Plus 6.0 software (Media Cybernetics, Rockville, MD, USA) [27]. Immunohistochemical (IHC) staining was performed to determine Bcl-2 and Bax expression. The Bcl-2 or Bax-positive area and total area were measured using Image Pro-Plus 6.0 software (Media Cybernetics). The percentage of positive area in the total area was used to quantify Bcl-2 or Bax expression levels. All analyses were performed by investigators who were blinded to the study group.

### 2.9. TUNEL Staining

The rate of apoptosis of articular chondrocytes was assessed using the TUNEL cell apoptosis detection kit (Servicebio, China), as described by the manufacturer. The specimens were viewed under a fluorescence microscope (Olympus, Japan), and the number of apoptotic articular chondrocytes was quantified in relation to the overall cell count.

### 2.10. Statical Analysis

All statistical analyses were implemented in SPSS v 22.0 (IBM SPSS Statistics for Windows; IBM Corp). Data are presented as mean ± standard deviation (SD). Differences among groups were determined using the *t*-test to compare significant differences. All immunoblots were performed in triplicate and one representative experiment is shown. Statistical significance was set at *P* < 0.05.

## 3. Results

### 3.1. Effect of OMT on Viability of Chondrocytes

**Figure 1:**
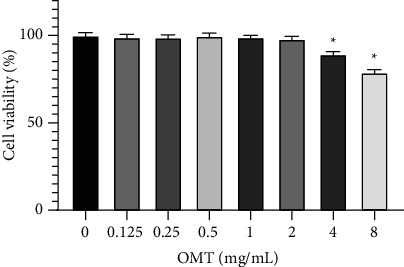
Effect of different concentration OMT on viability of chondrocytes. ^*∗*^*P* < 0.05 vs 0 mg/ml group OMT, oxymatrine.

The results of the CCK-8 cell viability assay revealed that OMT at 0–2 mg/mL had no conspicuous cytotoxicity on chondrocytes after 24 hours of incubation (Figure 1). OMT at concentrations of 4 and 8 mg/mL markedly reduced chondrocyte viability (Figure 1). Therefore, 0.5, 1, and 2 mg/mL OMT were used as the low, medium, and high doses, respectively, for subsequent experiments.

### 3.2. Effect of OMT on Genes Involved in Extracellular Matrix Metabolism of Chondrocytes

**Figure 2:**
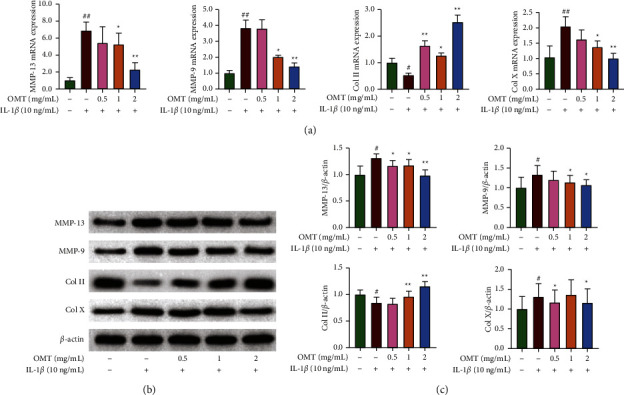
Effect of OMT on genes involved in extracellular matrix metabolism of chondrocytes. (a) The mRNA expression level of MMP-13, MMP-9, Col II, and Col (X) (b) and (c) Western blot results of MMP-13, MMP-9, Col II, Col X ^*∗*^*P* < 0.05 and ^*∗∗*^*P* < 0.01 vs IL-1*β*-induced group; ^#^*P* < 0.05 and ^##^*P* < 0.01 vs control group. IL, interleukin; MMP, matrix metalloproteinase; Col, Collagen; OMT, oxymatrine.

Next, we evaluated the effect of OMT on genes involved in extracellular matrix metabolism in IL-1*β* induced chondrocytes. The data showed that IL-1*β* stimulation mediated a significant upregulation of mRNA and protein expression of MMP13, MMP9, and Col X, but downregulated those of Col II in cultured chondrocytes (Figure 2(a) and 2(b)). However, 1 and 2 mg/mL OMT partly rescued Col II expression and dampened IL-1*β*-triggered expression of MMP13, MMP9, and Col X in chondrocytes (Figures 2(a) and 2(b)). These results suggest that OMT treatment can suppress the IL-1*β*-induced imbalance in extracellular matrix metabolism.

### 3.3. OMT Inhibits IL-1*β*-Triggered Apoptosis in Chondrocytes

**Figure 3:**
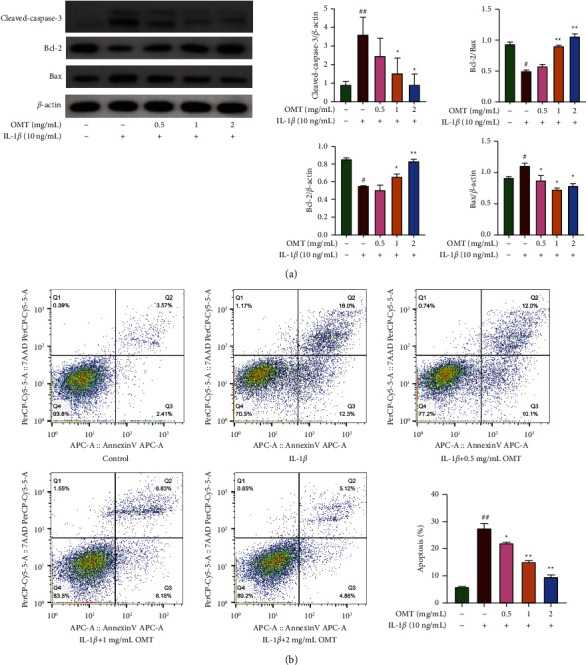
Impact of OMT on the IL-1*β*-triggered apoptosis in chondrocytes. (a) Western blot results of cleaved-caspase-3, Bcl-2, Bax. (b) Results of flow cytometry.^*∗*^*P* < 0.05 and ^*∗∗*^*P* < 0.01 vs IL-1*β*-induced group; ^#^*P* < 0.05 and ^##^*P* < 0.01 vs control group.

To investigate the effect of OMT on the mitochondrial pathway of apoptosis, apoptosis-linked factors and proapoptosis factors, specifically cleaved-caspase 3 and Bax, and the antiapoptotic factor Bcl-2, were quantified using western blots. Western blot results demonstrated that IL-1*β* treatment markedly upregulated synthesis of cellular apoptosis-related proteins including, cleaved-caspase-3 and Bax. Moreover, synthesis of Bcl-2 was downregulated, whilst OMT treatment reversed this effect in a dose-dependent manner (Figure 3(a)).

The effect of OMT on IL-1*β*-triggered apoptosis in chondrocytes was studied using flow cytometry. Results showed that IL-1*β* treatment markedly increased the number of apoptotic cells relative to that of the control groups, whilst OMT treatment remarkably reduced apoptosis of IL-1*β*-triggered chondrocytes in a dose-dependent manner (Figure 3(b)). Taken together, these data illustrate that OMT effectively dampens the apoptosis of IL-1*β*-triggered chondrocytes, and thus may have a protective role in OA development.

### 3.4. OMT Represses the NF-*κ*B Signaling Cascade in IL-1*β*-Triggered Chondrocytes

**Figure 4:**
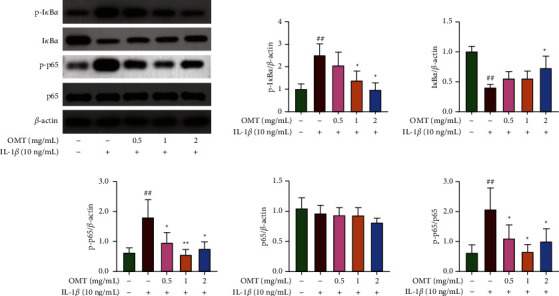
Impact of OMT on the IL-1*β*-triggered activation of the NF-*κ*B signaling pathway in chondrocytes. Ratios of p-I*κ*B*α*/I*κ*B*α* and p-p65/p65, ^*∗*^*P* < 0.05 and ^*∗∗*^*P* < 0.01 vs IL-1*β*-induced group; ^##^*P* < 0.01 vs control group.

Western blotting was used to evaluate the influence of OMT on the activation of the NF-*κ*B signaling cascade in IL-1*β*-triggered chondrocytes. Results showed IL-1*β* markedly enhanced phosphorylation of p65, while OMT remarkably attenuated phosphorylation of p65 triggered by IL-1*β* (Figure 4). Furthermore, IL-1*β* markedly upregulated the phosphorylation and degradation of I*κβα*, which was reversed by OMT in a dose-dependent manner (Figure 4). Collectively, these data demonstrate that OMT suppressed IL-1*β*-triggered NF-*κ*B signaling activation in chondrocytes *in vitro*.

### 3.5. OMT Inhibits Progression of OA in ACLT Mice

**Figure 5:**
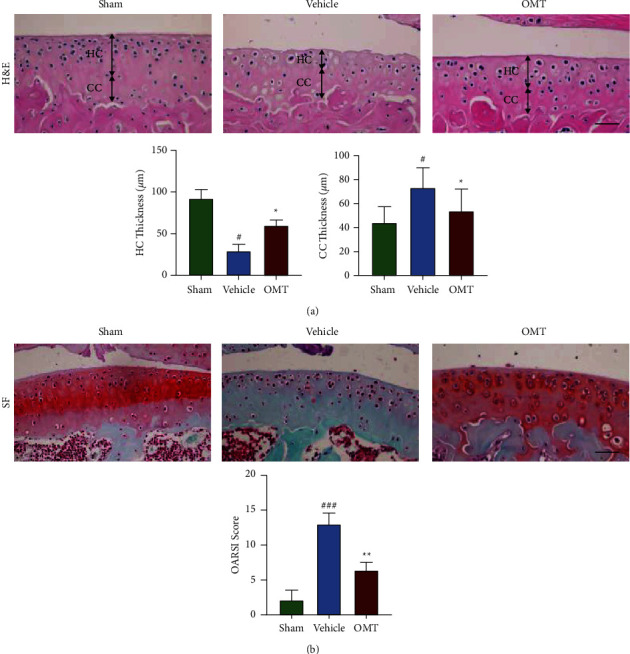
Hematoxylin-eosin (H&E) and Safranin O/Fast green (SF) staining show the effect of OMT on the progression of ACLT-triggered OA mice. (a) H&E results of mice in each group 8 weeks following surgery. Scale bar: 100 *μ*m. Below are the results of statistical analyses of HC and CC thickness. (b) SF staining results of mice in each group 8 weeks following surgery. Scale bar: 200 *μ*m. Below are the results of statistical analyses of OARSI articular cartilage score ^*∗*^*P* < 0.05 and ^*∗∗*^*P* < 0.01 vs vehicle group; ^#^*P* < 0.05 and ^###^*P* < 0.001 vs sham group.

**Figure 6:**
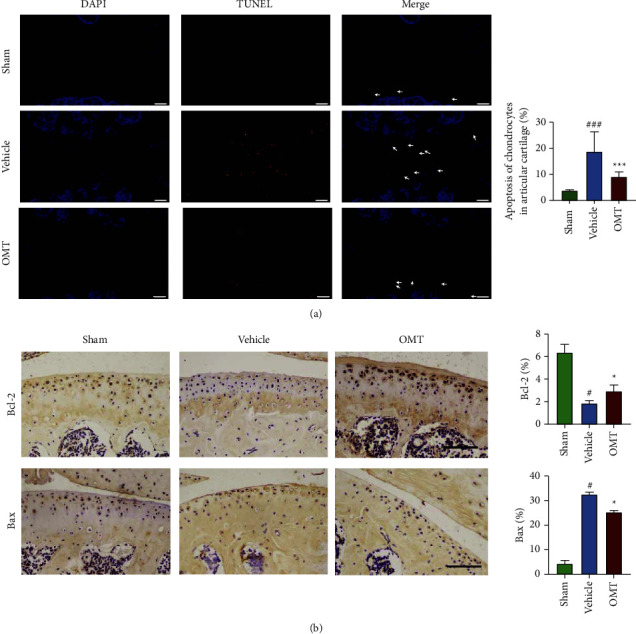
TUNEL and IHC staining for BCL-2 and Bax show the effect of OMT on progression of ACLT-triggered OA mice. (a) TUNEL results of mice in each group 8 weeks following surgery. Scale bar: 100 *μ*m. Right: results of statistical analyses of apoptotic chondrocyte rate. (b) IHC staining for BCL-2 and Bax expression of mice in each group 8 weeks following surgery. Scale bar: 100 *μ*m. Right: results of statistical analyses displayed as the percentage of Bcl-2 or Bax-positive expression area within the total area. ^*∗*^*P* < 0.05, and ^*∗∗∗*^*P* < 0.001 vs vehicle group; ^#^*P* < 0.05 and ^###^*P* < 0.001 vs sham group.

H&E (Figure 5(a)) and SF (Figure 5(b)) staining showed a smooth articular cartilage surface, stained red, in the sham group. Mice in the vehicle group showed severe destruction, erosion, and lesions of the articular cartilage, as well as increased calcified cartilage and vast loss of proteoglycans. Notably, OMT treatment reversed this effect. Furthermore, analysis showed that OMT treatment increased the IL-1*β*-triggered reduction of HC thickness (Figure 5(a)) and decreased IL-1*β*-triggered enhancement of HC thickness (Figure 5(a)) and OARSI score (Figure 5(b)). TUNEL staining showed significant chondrocyte apoptosis in the vehicle group compared with that in the sham group (Figure 6(a)). However, OMT markedly suppressed apoptosis in articular chondrocytes relative to the vehicle-treated group (Figure 6(a)). Moreover, OMT treatment increased the IL-1*β*-triggered downregulation of BCL-2 levels (Figure 6(b)) and decreased the IL-1*β*-triggered upregulation of Bax levels (Figure 6(b)). In summary, OMT exerted a protective effect against the degradation of articular cartilage in ACLT-induced OA mice.

## 4. Discussion

During the inflammatory response in OA, proinflammatory cytokines, such as IL-1*β*, inhibit the synthesis of aggrecan in chondrocytes and promote the expression of matrix metalloproteinases, thereby causing the articular cartilage to lose its normal structure and degrade the cartilage matrix [28]. Therefore, IL-1*β* is often used to induce chondrocyte injury in vitro [29]. In this study, we established that OMT remarkably reduced the imbalance of IL-1*β*-triggered extracellular matrix metabolism and cellular apoptosis in chondrocytes. The results from our *in vivo* experiments showed that OMT exerted a chondroprotective effect in ACLT-triggered OA mice. Previous studies [30] have used LPS in *in vitro* OA models to investigate the effect of OMT on chondrocytes. Although the cell models are different, the results of a previous study [30] and our present study both showed that OA exerts protective effects. OMT treatment may therefore be a promising chondroprotective approach to delay OA cartilage progression.

Homeostasis in the articular cartilage is pivotal to joint health [31]. Previous studies have revealed the importance of chondrocytes in the maintenance of articular cartilage homeostasis [4, 5]. In healthy cartilage tissue, chondrocytes maintain the necessary anabolic-catabolic balance for matrix maintenance and tissue function [32]. However, excessive mechanical stress and inflammatory conditions in OA promote catabolic activity during anabolism, thereby initiating cell death [33]. Previous studies have shown that MMPs play critical roles in joint degeneration by degrading extracellular matrix components, including cleavage of aggrecan and type II collagen, the main components of normal articular cartilage [34]. Under OA conditions, chondrocytes undergoing hypertrophy-like changes promote OA progression and upregulate the expression of catabolism-related cytokines [35]. Expression of type X collagen and MMPs in hypertrophic chondrocytes increases significantly, while synthesis of type II collagen decreases, and the metabolic activity of chondrocytes is unbalanced [36]. The results of the present study are consistent with previous studies demonstrating that IL-1*β* can induce the expression of MMP-9, type X collagen, and MMP-13 in chondrocytes [37, 38]. Moreover, we found that OMT partially rescued the imbalance between anabolic and catabolic activities in chondrocytes triggered by IL-1*β*, thereby promoting metabolic homeostasis.

Previous studies have associated OA pathogenesis with apoptosis-triggered chondrocyte death [39]. Notably, apoptosis induction in chondrocytes can be triggered by several stimuli, including increased tumor necrosis factor-*α* and IL-1*β* levels [40]. Previous studies have demonstrated that increased IL-1*β* upregulates proapoptotic cytokines such as Bax, but downregulates antiapoptotic cytokines such as Bcl-2 [39]. Consequently, these phenomena cause a decrease in the Bcl-2/Bax ratio, which ultimately leads to cellular apoptosis of chondrocytes [41, 42]. These results support findings from previous studies [39], illustrating the rate of apoptosis in OA chondrocytes to be remarkably higher than that of normal chondrocytes. Moreover, our results demonstrated that OMT inhibited expression of cleaved-caspase-3, Bax, and cleaved-caspase-9. Expression was promoted by IL-1*β* and upregulated by Bcl-2. Taken together, these findings indicate that OMT exhibits antiapoptotic properties through the mitochondrial apoptotic pathway. These results were partially confirmed by the *in vivo* experiments of this study. Specifically, articular chondrocytes from vehicle-treated mice exhibited markedly increased levels of cellular apoptosis than those of the sham group. However, OMT treatment markedly suppressed apoptosis of articular chondrocytes and degeneration of articular cartilage. Collectively, these results indicated that OMT can effectively delay OA progression in ACLT-induced OA mouse models.

The NF-*κ*B signaling cascade plays an indispensable role in stress and inflammatory responses as well as cell survival, especially during OA development [43, 44]. Previous investigations have shown that inhibitors of NF-*κ*B can effectively downregulate the expression of catabolic-related cytokines, which are triggered by IL-1*β* in chondrocytes. Results from *in vivo* experiments showed markedly lower levels of cartilage degeneration in the p65 knockout OA mouse model relative to the wild-type. Moreover, mounting evidence has revealed that NF-*κ*B can be activated in chondrocytes by various stimuli, including mechanical stress and injury-triggered inflammatory cytokines, increasing phosphorylation of p65 and I*κ*B, promoting catabolic changes and cellular apoptosis [45–47]. The results from the present study showed IL-1*β* promoted cellular apoptosis and catabolic activities in chondrocytes by activating the NF-*κ*B signaling cascade. Notably, OMT intervention effectively inhibited the expression of the IL-1*β*-triggered NF-*κ*B signaling cascade by suppressing phosphorylation of p65 and I*κ*B, findings which are consistent with those of previous studies [20–22]. We therefore speculated that OMT may play a protective role in chondrocytes during OA development by inhibiting the NF-*κ*B signaling cascade. Other signals may also be involved in the action of OMT. Since we did not add an NF-*κ*B activator to block the action of OMT, we could not confirm this, though this could be a direction for future work.

## 5. Conclusion

Taken together, OMT can decrease IL-1*β*-induced apoptosis and ECM metabolic imbalance in chondrocytes *in vitro*, exerting a protective effect on the degradation of articular cartilage in ACLT-triggered OA mice. These results suggest OMT is a protective factor against chondrocyte injury during OA. OMT may also play a role in chondrocytes during OA through inhibition of NF-*κ*B signaling through the reduction of phosphorylation of p65 and I*κ*B. Thus, OMT may be a potential chondroprotective agent for delaying OA progression.

## Figures and Tables

**Table 1:** Primer sequence.

MMP-13	Forward	TGTTTGCAGAGCACTACTTGAA
	Reverse	CAGTCACCTCTAAGCCAAAGAAA

MMP-9	Forward	GCAGAGGCATACTTGTACCG
	Reverse	TGATGTTATGATGGTCCCACTTG

Col II	Forward	GGGAATGTCCTCTGCGATGAC
	Reverse	GAAGGGGATCTCGGGGTTG

Col X	Forward	TTCTGCTGCTAATGTTCTTGACC
	Reverse	GGGATGAAGTATTGTGTCTTGGG

*β*-actin	Forward	GTGACGTTGACATCCGTAAAGA
	Reverse	GCCGGACTCATCGTACTCC

## Data Availability

The datasets generated and analyzed in the present study are available from the corresponding author upon reasonable request.

## Additional Points

All figures submitted have been created by the authors, who confirm that the images are original with no duplication and have not been previously published in whole or in part.

## Supplementary Materials

Supplementary Materials Supplementary Table 1: the results of CCK-8. Supplementary Table 2: the results of flow cytometry.
