# Supplementary figures and images for: Oxymatrine Protects Chondrocytes against IL-1β-triggered Apoptosis in Vitro and Inhibits Osteoarthritis in Mice Model
Source: Evid Based Complement Alternat Med. 2022 Sep 27;2022:2745946. doi: 10.1155/2022/2745946 (PMC9532098; doi:10.1155/2022/2745946)

Supplementary-Flow cytometry raw data


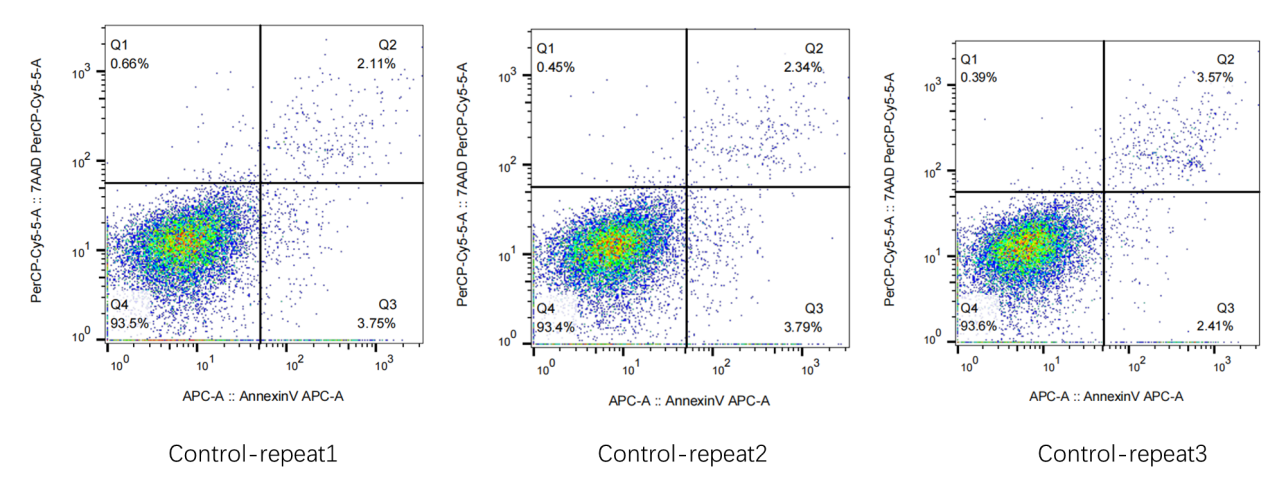


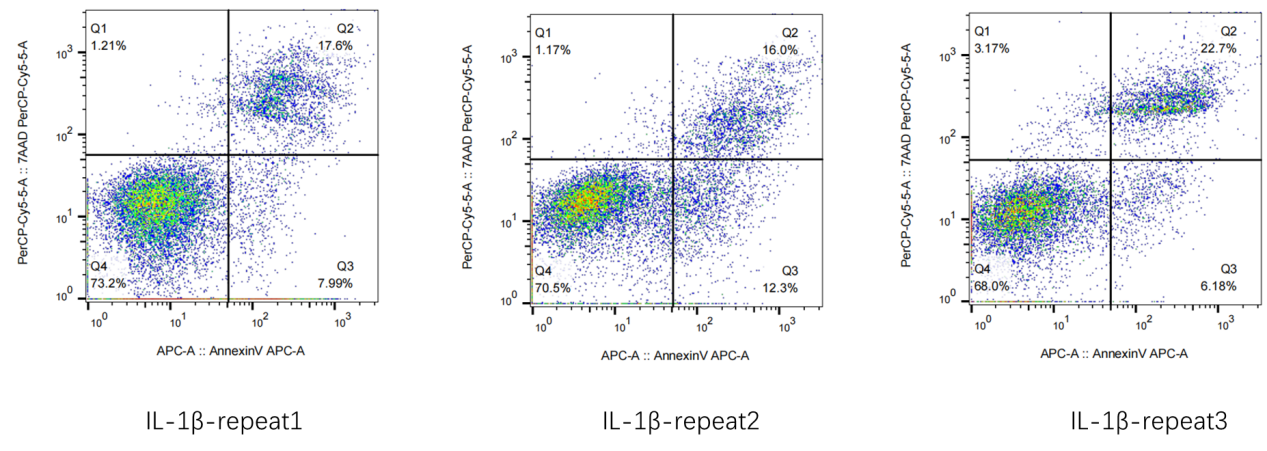


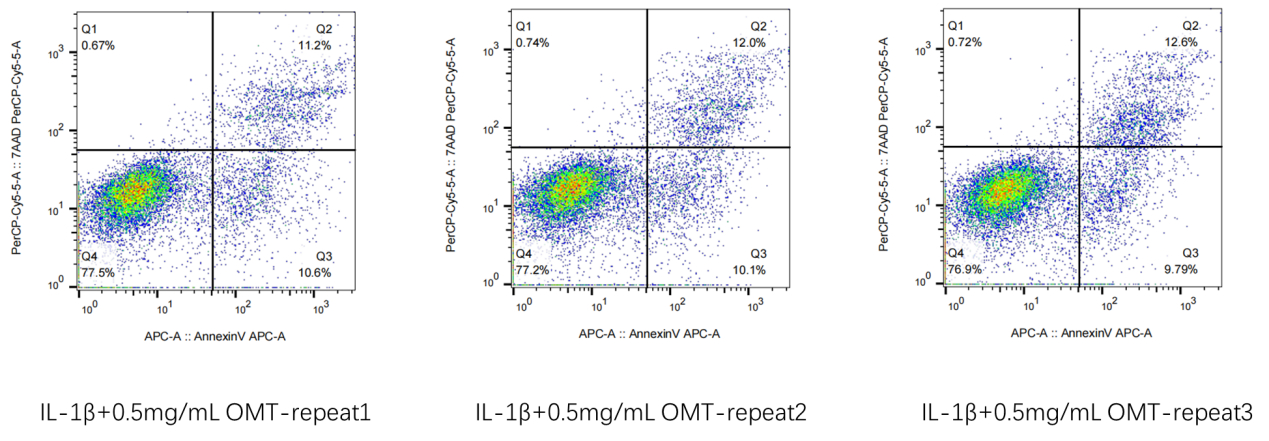


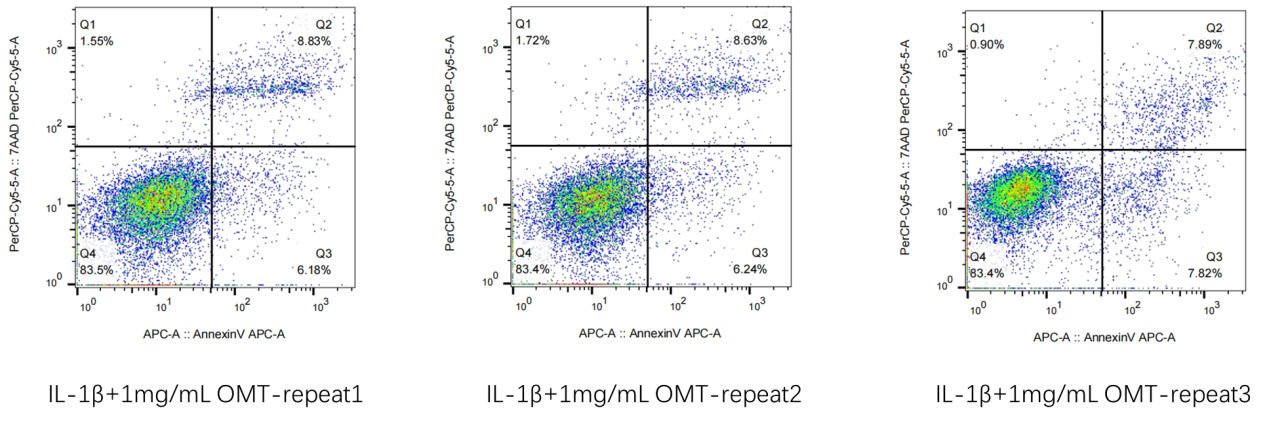


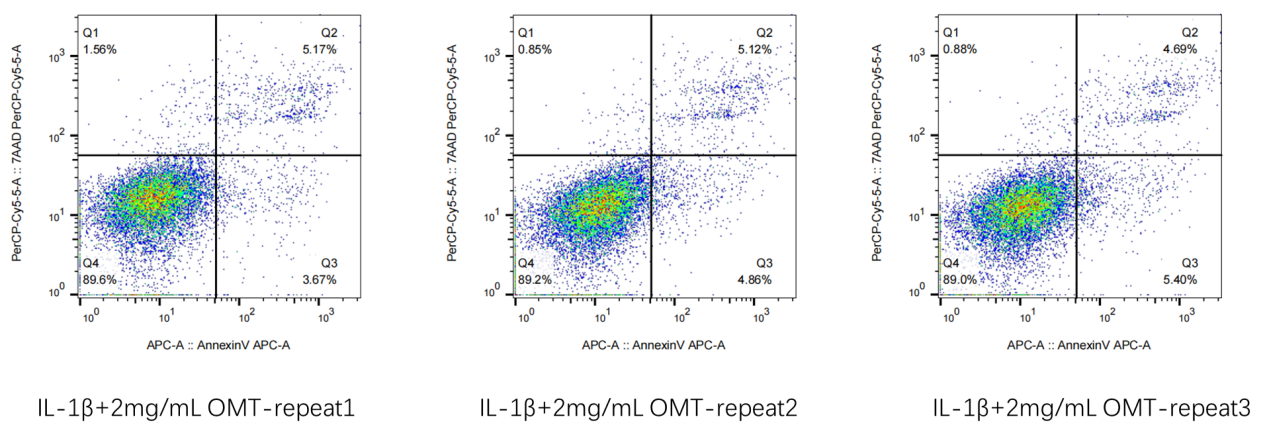


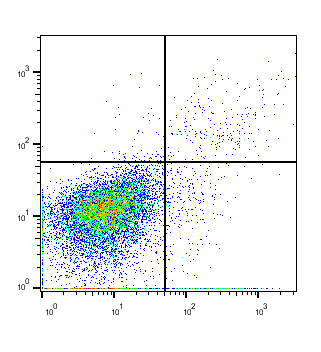

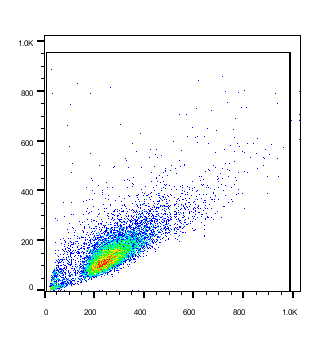


1. **Gate strategy**
2. **FSC-A**
3. **SSC-A**
4. **Annexin V**
5. **7AAD**

Supplement: Supplementary Materials — Supplementary Table 1: the results of CCK-8. Supplementary Table 2: the results of flow cytometry. [file 2745946.f1.zip › supplementary table 2.docx]
